# Fast globalized parameter tuning of antennas using simplex predictors, multilevel EM simulations and principal directions

**DOI:** 10.1038/s41598-025-21899-2

**Published:** 2025-10-31

**Authors:** Anna Pietrenko-Dabrowska, Slawomir Koziel

**Affiliations:** 1 Faculty of Electronics, Telecommunications and Informatics, Gdansk University of Technology, Gdansk, 80-233 Poland; 2 Engineering Optimization & Modeling Center, Reykjavik University, Reykjavik, 102 Iceland

**Keywords:** Antenna design, CAD, EM-driven design, Global optimization, Simplex, Surrogate modeling, Variable-fidelity models, Principal directions, Electrical and electronic engineering, Computational science

## Abstract

Rigorous numerical optimization is ubiquitous in modern antenna design. In many cases, performing local (e.g., gradient-based) parameter tuning is sufficient. However, a global optimization is necessary in many practical scenarios, which incurs tremendous computational expenses and is often unmanageable, mainly when carried out using electromagnetic (EM) analysis. It is possible to mitigate this issue using surrogate modeling techniques. Still, building quality metamodels is hindered by the curse of dimensionality and the necessity of setting up the models across extended ranges of geometry parameters. This paper presents a cost-effective technique for globalized antenna optimization. Our approach carries out the search process in the space of antenna operating parameters (e.g., center frequencies), using simplex-based regression predictors and variable-resolution EM simulations. The stage of global optimization, conducted using low-fidelity EM analysis, is complemented by rapid gradient-based tuning performed using high-resolution models. The latter is accelerated by performing the antenna sensitivity only along certain (principal) directions, affecting response variability to the greatest extent. Comprehensive validation using four microstrip antennas shows the excellent performance of the presented method and its superior computational efficiency over several benchmark methods (less than eighty high-fidelity EM simulations are required to render optimal design on average).

## Introduction

 Emerging application areas (5G/6G technologies^1,2^, space communications^3^, internet of things^4^, vehicle radar systems^5^, energy scavenging^6^, medical imaging^7^ require new types of antennas, equipped with appropriate functionalities (broadband, multi-band operation^8–10^, reconfigurability^11^, gain enhancement^12^, beam steering^13^, and—in many cases—occupying limited physical space^14–16^. Satisfying these demands leads to geometrically involved topologies that require full-wave electromagnetic (EM) analysis to be properly evaluated. At the same time, their geometry parameters must be thoroughly adjusted to secure the best possible performance. Traditionally used methods, primarily based on supervised parametric studies, are grossly insufficient in this respect. Instead, rigorous numerical optimization is recommended^17–19^ despite being CPU-intensive, even when performing local tuning. Global optimization^20–22^ (also multi-objective design^23–25^ is incomparably more expensive yet necessary in many practical situations. Examples include the design of metasurfaces^26–28^, simulation-based miniaturization^29^, array pattern synthesis^30^, unavailability of a good starting point^31^, or large-scale antenna dimension scaling (e.g., w.r.t. the operating frequencies^32^.

Global optimization predominantly uses nature-inspired techniques^33–41^. Some popular methods include particle swarm optimization (PSO), firefly optimization, grey wolf algorithm, differential evolution (DE), or harmony search, e.g^42–46^. , . New methods, often featuring fancy names, have been emerging at accelerated rates^47–53^. The ability to avoid getting stuck in inferior local solutions is arguably the effect of information exchange between the candidate solutions, either in the form of the operators changing the parameter vector content (crossover, mutation^54^ or by biasing its relocation within the parameter space (e.g., towards the most promising solution identified thus far^55^. Perhaps the most attractive feature of bio-inspired routines is their simplicity^56^. The drawback is inferior computational efficiency, typically measured in thousands of fitness function calls per run. Due to this, direct antenna optimization is only feasible if analytical models are available (e.g., pattern synthesis using array factor models^57,58^, which, however, fail to accommodate mutual coupling phenomenon^59^, EM analysis is fast (simple structures, coarse discretization), or available resources permit parallelization.

Practical global optimization is usually conducted by employing surrogate-assisted procedures^60–66^ (e.g., Gaussian process regression, kriging, support vector regression, neural networks). Yet, constructing accurate data-driven models requires considerable budgets in training data acquisition, especially for multi-parameter structures. A workaround has been offered by machine learning frameworks^67,68^, in which the metamodel is used as a predictor while being iteratively enhanced with the use of EM-simulated samples acquired throughout the search process^69–72^. Physics-based surrogate models (space mapping^73^, adaptive response correction^74^, cognition-driven design^75^ can be utilized as well, but they are mainly predestined for local optimization purposes.

The primary limitation of surrogate-assisted algorithms is a rendition of reliable data-driven models, partly due to dimensionality-related problems but also the necessity of representing multiple nonlinear characteristics (reflection, gain, axial ratio) over broad ranges of frequency and geometry parameters. Consequently, most reported procedures are showcased using relatively simple problems^76,77^. Possible mitigation methods include constrained modeling techniques^78–83^, which focus model identification on the regions encapsulating high-quality designs; the latter can be identified using pre-sampling or the employment of pre-optimized reference designs^81^. An additional alternative is the response feature methodology^84,85^, which is founded on restating the problem regarding the so-called feature points of the antenna outputs (e.g., the resonances). This enables regularization of the merit function landscape, eventually speeding up optimization algorithms^86,87^ but also reducing the sizes of the datasets required for building surrogate models^88^.

This work aims to introduce a procedure for globalized antenna optimization, which provides a performance edge over available techniques. To achieve low running costs, the search process is executed using regression models in the form of simplexes built from the viewpoint of the operating parameters of the considered antenna. The latter inherently regularizes the objective function, facilitating and expediting optimum identification. For further acceleration, the initial optimization stage is carried out using low-resolution EM simulations and terminated using relatively loose criteria concerning the compliance of the actual and required antenna operating parameters. The search process is complemented by local, gradient-based parameter tuning. To ensure reliability, it is performed using high-resolution EM analysis. However, finite-differentiation sensitivity updates are only applied along selected directions identified as having the major effects on antenna response variability; this approach leads to substantial cost reduction without being detrimental to design quality. Validation of our technique includes four antenna structures of distinctive characteristics. The superior efficacy of the developed approach over benchmark techniques concerning reliability, solutions’ repeatability, and cost efficacy has been demonstrated. The average cost corresponds to only about eighty EM simulations at a high resolution, which is remarkable, considering the globalized search capability of the discussed framework.

The key technical contributions are outlined below: (i) the development of a globalized antenna optimization procedure involving low-cost regression models targeting antenna operating parameters, (ii) the incorporation of several acceleration mechanisms, including problem reformulation (in a feature-based-like fashion), variable-resolution EM simulations, and restricted sensitivity updates using principal directions, (iii) the development of convergence-guaranteeing mechanisms both at the global and local search stages, (iv) the implementation of a complete algorithmic framework employing the listed components, (v) demonstration of the effectiveness of the technique with regard to reliability, solution repeatability, and remarkable computational efficiency.

## Globalized search by regression predictors, variable-resolution models, and restricted sensitivity updating

The details of the presented optimization procedure are provided in this part of the paper. We start by discussing the way the design task is posed (Sect. 2.1), variable-resolution EM simulations (Sect. 2.2), and the definition and properties of regression-based surrogates (Sect. 2.3). Section 2.4 delineates the global search stage. Section 2.5 describes local parameter tuning along with the acceleration mechanisms implemented therein. The complete procedure is outlined in Sect. 2.6.

### Problem formulation

The optimization aims to adjust the vector $\boldsymbol{x} = \left[ {x_{1} \ldots x_{n} } \right]^{T}$ of antenna decision variables to minimize cost function *U* that evaluates the design merit. The optimal design x^*^ is therefore identified as

1 $$\boldsymbol{x}^{*} = \arg \mathop {\min }\limits_{\boldsymbol{x}} U(\boldsymbol{x},\boldsymbol{f}_{t})$$

where target vector of operating frequencies is denoted as $\boldsymbol{f}_{t} = \left[ {f_{{t.1}} \ldots f_{{t.K}} } \right]^{T}$ (*K* being the number of antenna center frequencies or bands). When constrained tasks are considered, computationally cheap constraints (e.g., pertinent to antenna size) are treated explicitly. In contrast, the expensive ones (requiring EM analysis for their evaluation) are assumed to be treated implicitly by means of penalty functions^97^.

The expression (1) distinguishes the operating frequencies to underscore that their appropriate arrangement is the critical difficulty in global optimization. Our algorithm employs quantification of the displacement of the actual operating frequencies with regard to the targets as the principal measure of design quality. Consequently, the notation introduced above is crucial for further consideration.

### Dual-resolution EM analysis

Variable-fidelity models were utilized in antenna design for quite some time to accelerate EM-driven design procedures^89^. Typically, lower-fidelity representations are achieved by lowering the structure’s discretization density, which speeds up the simulation (by a factor from three to ten depending on the antenna structure), compromising accuracy (other options include neglecting losses, reducing computational domain, etc.)^90,91^. Practical design procedures typically use two resolution levels (coarse/fine, low/high)^92^. Recently, model management schemes involving a continuous range of resolutions have been proposed^93,94^. Often, the low-fidelity model needs to be refined before being employed (e.g., space mapping^95^, although, in some applications, it can be used raw (e.g., parameter space pre-screening^96^. In this work, two resolution levels are used: the low-resolution model ***R***_*c*_(***x***), and the high-resolution model ***R***_*f*_ (***x***). ***R***_*c*_(***x***) is employed in the global search stage (Sect. 2.3 and 2.4); ***R***_*f*_ (***x***) is applied in the final tuning step discussed in Sect. 2.5.

### Simplex-based regression models

Global EM-driven antenna optimization is extremely costly, which is this work’s main point of interest. Surrogate-assisted techniques can mitigate this problem^60–68^; however, constructing reliable behavioral models over large spaces is problematic. The reflection responses simulated at randomly allocated designs across the design space indicate significant variability, which makes local parameter tuning unreliable if the starting point is away from the optimal one. Meanwhile, establishing an accurate data-driven surrogate representing highly nonlinear frequency characteristics necessitates increased amounts of training data and may even be impossible if parameter ranges are broad. On the other hand, the situation becomes incomparably simpler when the perspective of antenna operating parameters is taken. The relationships between, e.g., antenna resonant frequencies and their geometrical parameters are regular (essentially monotonic) even for random observables (not optimized). This type of relationship is common for high-frequency devices^85–88^.

The developed optimization procedure explores the properties outlined in the previous paragraph. The simplicity of the discussed relationship makes it sufficient to carry out the search process using low-complexity surrogates representing the antenna operating parameters rather than complete frequency responses. To account for the parameter space dimensionality *n*, one must involve at least *n* linearly independent directions (or *n* + 1 affinely independent vectors ***x***^(*j*)^). From this point of view, the appropriate geometric object is a simplex. The remainder of this section defines the simplex-based models. We use the notation provided in Tab 1. In our approach, operating figure vectors are used, typically comprising antenna centre frequency (frequencies) or substrate relative permittivity. We also employ performance vectors ***l*** serving for additional assessment of design quality (other than that quantified by ***f***). For instance, consider a dual-band antenna where the operating figure vector may comprise antenna centre frequencies ***f*** = [*f*_1_ *f*_2_]^*T*^ and the vector of performance figures may comprise values of reflection at operational frequencies ***l*** = [*l*_1_ *l*_2_]^*T*^. Another example includes a quasi-Yagi antenna (see Sect. 3), for which we have ***f*** = *f*_1_, and ***l*** = [*l*_1_ *l*_2_]^*T*^ (*l*_1_ equals reflection at *f*_1_, and *l*_2_ is maximum realized gain).

Let’s assume that are affinely independent vectors in the design space *X* (which is bounded by lower and upper limits for the parameters, i.e., antenna dimensions), whereas be the associated operating and performance figure vectors, respectively. In practice, the elements of {***x***^(*j*)^}_*j* = 0, …, *n*_, are found via random sampling, extraction of ***f***(***x***^(*j*)^) and ***l***(***x***^(*j*)^) from EM simulation data, and approving vectors that fit into the limits listed in Table 1.

**Table 1:** Regression models in the form of simplexes: notation.

	Operating figures	Performance figures
Symbol	***f*** = [*f*_1_ … *f*_*N*_]^*T*^	***l*** = [*l*_1_ … *l*_*M*_]^*T*^
Explanation	Vector comprising antenna centre frequency/frequencies or substrate relative permittivity	Vector comprising quantities determining design quality (not present in in the vector ***f***), such as reflection levels, gain, axial ration at specific frequencies or bands
Symbol	***f***_*L*_ = [*f*_*L*.1_ … *f*_*L.N*_]^*T*^, ***f***_*U*_ = [*f*_*U*.1_ … *f*_*U.N*_]^*T*^	***l***_*L*_ = [*l*_*L*.1_ … *l*_*L.M*_]^*T*^, ***l***_*U*_ = [*l*_*U*.1_ … *l*_*U.M*_]^*T*^
Explanation	Lower/upper bounds, i.e., only vectors satisfying the conditions *f*_*L.j*_ ≤ *f*_*j*_ ≤ *f*_*U.j*_, *j* = 1, …, *N*, are of interest	Lower/upper bounds, i.e., only vectors satisfying the conditions *l*_*L.j*_ ≤ *l*_*j*_ ≤ *l*_*U.j*_, *j* = 1, …, *M*, are of interest Remark 1: we may have *l*_*L.j*_ = –∞, for some *j* (i.e., no lower bound for *l*_*j*_ exists) Remark 2: we may have *l*_*U.j*_ = ∞, for some *j* (i.e., no upper bound for *l*_*j*_ exists)

As the vectors $\boldsymbol{x}^{{\left( j \right)}} - \boldsymbol{x}^{{\left( 0 \right)}}$ are linearly independent, thus uniqueness of expansion is ensured

2 $$\boldsymbol{x} = \boldsymbol{x}^{(0)} + \sum\limits_{j = 1}^{n} {a_{j} (\boldsymbol{x}^{(j)} - \boldsymbol{x}^{(0)} )}$$

for any vector ***x*** ∈ *X*. The coefficients ***a*** = [*a*_1_ … *a*_*n*_]^*T*^ are identified as

3 $$\boldsymbol{a}(\boldsymbol{x}) = \boldsymbol{X}^{ - 1} (\boldsymbol{x} - \boldsymbol{x}^{(0)} )$$

with

4 $$X = \left[ {x^{(1)} - x^{(0)} \;\; \cdots \;\;\;x^{(n)} - x^{(0)} } \right]$$

is an invertible *n* × *n* matrix. The simplex surrogates ***F***(***x***) : *X* → *F*, and ***L***(***x***) : *X* → *R*^*M*^, representing the operating and performance vectors over *X* and *R*^*M*^, are then defined as

5 $$\boldsymbol{F}(\boldsymbol{x}) = \boldsymbol{f}^{(0)} + \sum\limits_{j = 1}^{n} {a_{j} } (\boldsymbol{f}^{(j)} - \boldsymbol{f}^{(0)} ) = \boldsymbol{f}^{(0)} + \boldsymbol{X}_{f} \boldsymbol{a}(\boldsymbol{x}) = \boldsymbol{f}^{(0)} + \boldsymbol{X}_{f} \boldsymbol{X}^{ - 1} (\boldsymbol{x} - \boldsymbol{x}^{(0)} )$$

6 $$\boldsymbol{L}(\boldsymbol{x}) = \boldsymbol{l}^{(0)} + \sum\limits_{j = 1}^{n} {a_{j} } (\boldsymbol{l}^{(j)} - \boldsymbol{l}^{(0)} ) = \boldsymbol{l}^{(0)} + \boldsymbol{X}_{l} \boldsymbol{a}(\boldsymbol{x}) = \boldsymbol{l}^{(0)} + \boldsymbol{X}_{l} \boldsymbol{X}^{ - 1} (\boldsymbol{x} - \boldsymbol{x}^{(0)} )$$

where ***a*** is computed using (3), and

7 $$\boldsymbol{X}_{f} = \left[ {\boldsymbol{f}^{(1)} - \boldsymbol{f}^{(0)} \;\;\; \cdots \;\;\;\boldsymbol{f}^{(n)} - \boldsymbol{f}^{(0)} } \right]$$

8 $$\boldsymbol{X}_{l} = \left[ {\boldsymbol{l}^{(1)} - \boldsymbol{l}^{(0)} \;\;\; \cdots \;\;\;\boldsymbol{l}^{(n)} - \boldsymbol{l}^{(0)} } \right]$$

At this point, a comment is necessary regarding the simplicity of the proposed regression models. Their sufficiency follows from using the operating parameters as the model outputs, rather than highly nonlinear antenna characteristics (reflection, gain, etc.). As demonstrated in the literature (e.g^106^. , , the relationships between operating parameters and design variables are weakly nonlinear. Consequently, the predictive power of simple regressors constructed using a limited number of training points is considerably better compared to conventional representations (with the model outputs being complete frequency characteristics, etc.). As extensively demonstrated in Sect. 3, this allows us to efficiently handle various types of responses, for example, reflection coefficient and gain.

### Global search stage

The global part of the optimization process utilizes ***F***(***x***) and ***L***(***x***), as defined in the previous section. For computational efficiency, it is executed using EM model of low fidelity ***R***_*c*_. The simple relationship between the operating and geometry parameters discussed in Sect. 2.3 makes ***F***(***x***) and ***L***(***x***) reliable predictors of the antenna performance. The search process arrangements are described below.

#### Design quality quantification

**Fig. 1:**
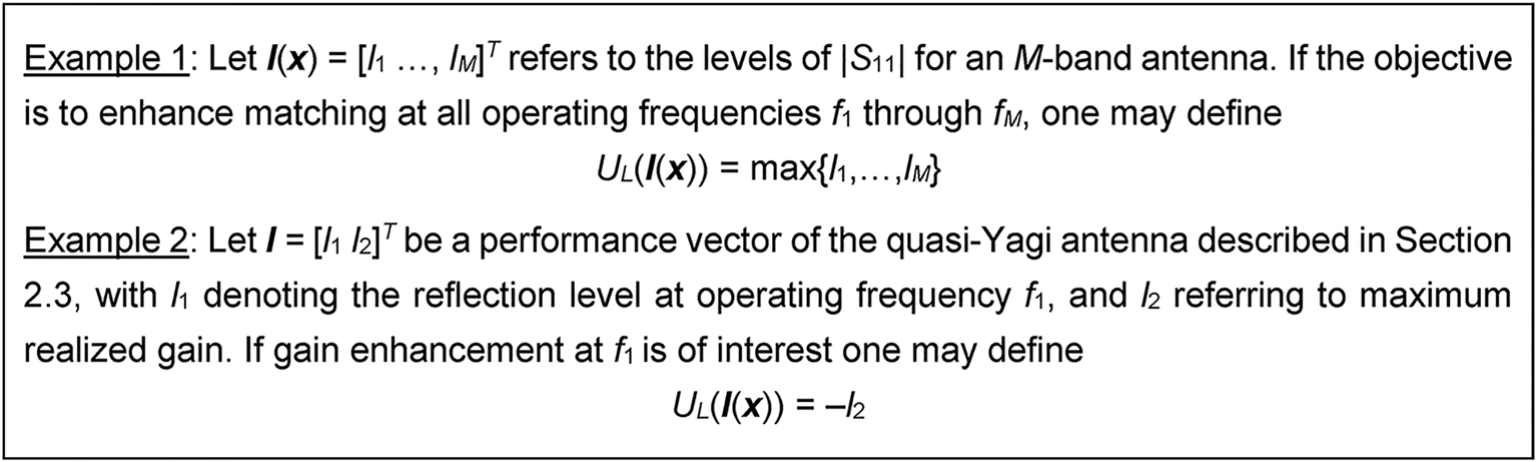
Examples of the cost function* U*_*L*_, cf. (9)

The design quality will be assessed using an objective function *U*_*F*_, which considers the vectors ***f***(***x***) and ***l***(***x***), which, in turn, refer to the operating and performance properties, respectively. Our primary objective at this process stage is to align ***f***(***x***) with the target ***f***_*t*_. For notational simplicity, the symbol ***f***_*t*_ indicates the vectors comprising both target operating figures and target operating frequencies (cf. Section 2.1). In our considerations, we use the following definition of the function *U*_*F*_

9 $$U_{F} (\boldsymbol{x}) = U(\boldsymbol{f}(\boldsymbol{x}),\boldsymbol{l}(\boldsymbol{x})) = U_{L} (\boldsymbol{l}(\boldsymbol{x})) + \beta_{F} ||\boldsymbol{f}(\boldsymbol{x}) - \boldsymbol{f}_{t} ||^{2}$$

in which *U*_*L*_ coincides with the original objective function *U*, although it is evaluated using ***l***(***x***) rather than the complete antenna characteristics. To clarify the matter, consider the examples shown in Fig. 1. It should be emphasized that it is not necessary for *U*_*L*_ to mimic *U* exactly. Again, at this stage, the goal is to ensure that ***f***(***x***) converges towards ***f***_*t*_. as much as possible, which is exacted by the regularization term of (9).

#### Global search procedure

The models ***F***(***x***) and ***L***(***x***) are used as predictors to identify the optimum design as defined by (11), i.e., minimizing of the merit function *U*_*F*_. The new optimum approximation is obtained as

10 $$\boldsymbol{x}_{tmp} = \arg \mathop {\min }\limits_{x \in X} U_{F} (\boldsymbol{F}(\boldsymbol{x}),\boldsymbol{L}(\boldsymbol{x}))$$

Note that ***f***(***x***) and ***l***(***x***) were replaced by the respective models ***F***(***x***) and ***L***(***x***). Having in mind that the model’s accuracy is the best in the vicinity of the simplex anchor points {***x***^(*j*)^}_*j* = 0,…,*n*_, the solution to (10) is subject to the constraints (recall that ***a***(***x***) are expansion coefficients defined by (3))

11 $$\sum\limits_{j = 1}^{n} {a_{j} = 1}$$

12 $$- \alpha \le a_{j} \le 1 + \alpha , \ldots j = 1,\, \ldots n$$

with *α* > 0 being a small number (e.g., *α* = 0.2). Both (11) and (12) restrict the search process to the simplex interior and its vicinity.

For further consideration, the simplex vertices are ordered w.r.t. decreasing values of ||***f***^(*j*)^ – ***f***_*t*_||. In particular, the norm corresponding to ***x***^(0)^ is the smallest one, and will be used as the starting point for solving (12). We also have ***a***(***x***^(0)^) = [0 … 0]^*T*^. The vector ***x***_*tmp*_ is only accepted if.

13 $$\left| {\left| {\boldsymbol{f}_{tmp} {-}\boldsymbol{f}_{t} } \right|} \right| \, = \, \left| {\left| {\boldsymbol{f}\left( {\boldsymbol{x}_{tmp} } \right) \, {-}\boldsymbol{f}_{t} } \right|} \right| \, < \max \{ j\in\left\{ {0, \, 1, \, \ldots ,n} \right\}:\left| {\left| {\boldsymbol{f}^{(j)} - \boldsymbol{f}_{t} } \right|} \right|\}$$

in which case ***x***_*tmp*_ replaces the worst-quality vertex ***x***^(*jworst*)^ with.

14 $$j_{worst} = \, \arg \max \{ j\hat{I}\left\{ {0, \, 1, \, \ldots ,n} \right\}:\left| {\left| {\boldsymbol{f}^{(j)} - \boldsymbol{f}_{t} } \right|} \right|\}$$

In any other case, ***x***_*tmp*_ is discarded, which is followed by simplex reduction towards ***x***^(0)^. We have.

15 $$\boldsymbol{x}^{(j)} \leftarrow \gamma \boldsymbol{x}^{(j)} + (1 - \gamma )\boldsymbol{x}^{(0)} \ldots for \ldots j = 1, \ldots ,n$$

**Fig. 2:**
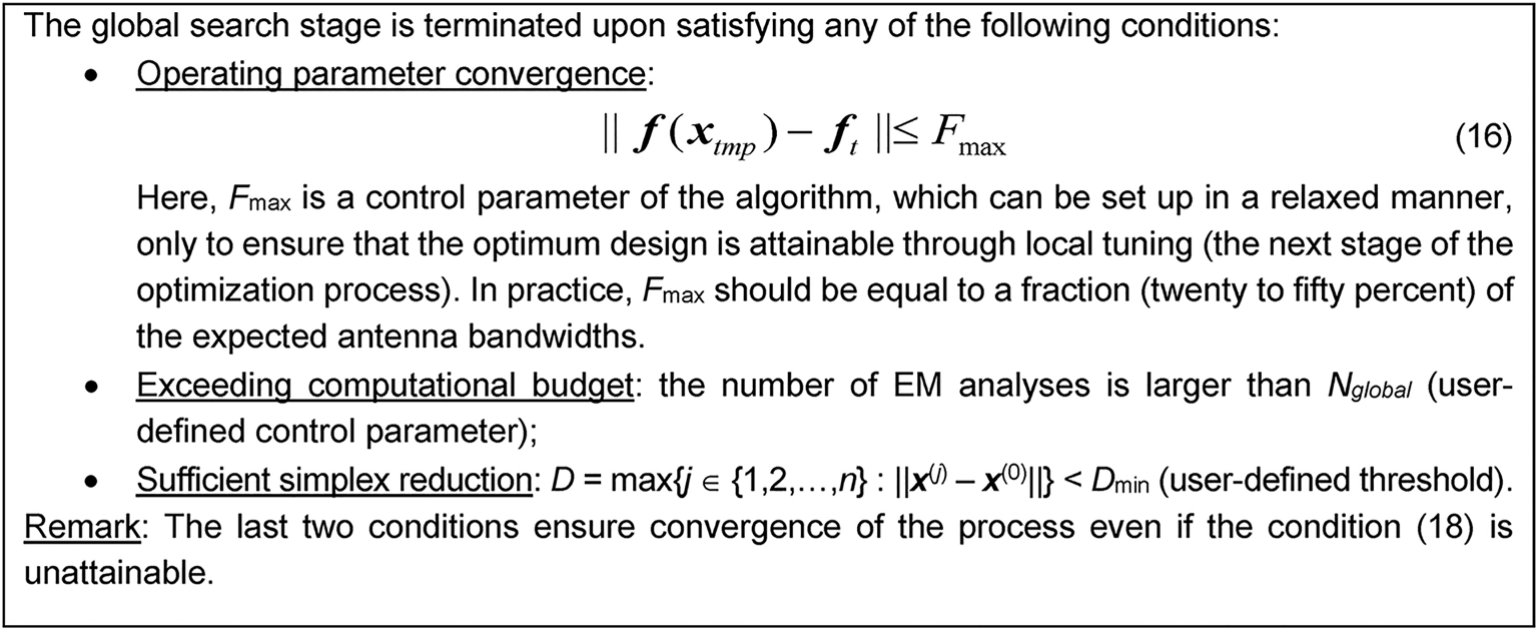
Termination conditions for the global search stage of the proposed optimization procedure.

The reduction factor is *γ* = 0.5 but this value is not critical. It can be demonstrated that sufficient simplex reduction necessarily improves the vertex quality by diminishing ||***f***^(*j*)^ – ***f***_*t*_||, which guarantees the satisfaction of the condition (13). The simplex is iteratively updated according to the rules mentioned until one of the termination conditions listed in Fig. 2 has been satisfied. For continuously differentiable functions ***f***(***x***) and ***l***(***x***), it is possible to show that a sufficient shrinkage of the simplex size necessarily improves the function *U*_*F*_ and the norm ||***f***(***x***) – ***f***_*t*_||.

### Rapid gradient-based local tuning with principal directions

The global search, terminated upon satisfying any of the conditions listed in, is followed by local tuning of design variables. The base search mechanism is the trust-region (TR) gradient-based routine with numerical derivatives^98^. This step is executed using the high-resolution EM model ***R***_*f*_.

Sensitivity estimation is the key factor driving computational expenses of the TR algorithm. Here, to reduce that overhead, the basic version of the TR procedure is enhanced by restricted sensitivity updating using principal directions^99^. The outline of the technique is provided below. The main idea is to limit finite differentiation to few directions that have a significant influence on antenna characteristics.

The first step is to determine an orthonormal basis of vectors {***v***^(*j*)^}_*j* = 1, …, *n*_, ordered with regard to their significance for the assumed antenna response variability metric *F*_*v*_. As the design specifications of the antenna are usually bounded to certain range of frequencies *F*, one might define *F*_*v*_ with these particular frequencies in mind. Let Then, we can define^100^

17 $$F_{v} \left( {\boldsymbol{R}_{1} (\boldsymbol{x}_{1} ),\boldsymbol{R}_{2} (\boldsymbol{x}_{2} )} \right) = \sqrt {\sum\limits_{{f_{k} \in F}} {\left[ {R_{1} (\boldsymbol{x}_{1} ,f_{k} ) - R_{2} (\boldsymbol{x}_{2} ,f_{k} )} \right]^{2} } }$$

where *F* refers to the considered frequency range (i.e., such that reflects design specifications. For the current iteration ***x***^(*i*)^ and the Jacobian matrix ***J***_*R*_(***x***^(*i*)^), we carry out maximization of *F*_*v*_ which yields the first principal direction ***v***^(1)^

18 $$\boldsymbol{v}^{{(1)}} = \arg \mathop {\max }\limits_{{\boldsymbol{v};\;||\boldsymbol{v}|| = 1}} F_{v} \left( {\boldsymbol{L}^{{(i)}} (\boldsymbol{x}^{{(i)}} + \boldsymbol{v}),\boldsymbol{R}(\boldsymbol{x}^{{(i)}} )} \right)$$

with ***L***^(*i*)^ being the linear model (17). Vectors ***v***^(2)^, ***v***^(3)^, …, are obtained similarly with additional conditions imposed to ensure that they form an orthonormal basis. Given $v^{{\left( k \right)}} ,k = 1, \ldots ,j,$ the vector ***v***^(*j*+1)^ is found as^100^

19 $$\boldsymbol{v}^{{(j + 1)}} = \arg \mathop {\max }\limits_{{\bar{v}}} F_{v} \left( {\boldsymbol{L}^{{(i)}} (\boldsymbol{x}^{{(i)}} + \bar{\boldsymbol{v}}),\boldsymbol{R}(\boldsymbol{x}^{{(i)}} )} \right)$$

where $\bar{\boldsymbol{v}} = P^{{(j)}} \left( \boldsymbol{v} \right)\left\| {P^{{(j)}} \left( \boldsymbol{v} \right)} \right\|$. The projection *P*^(*j*)^ is defined as $P^{{(j)}} \left( \boldsymbol{v} \right) = \boldsymbol{v} - \sum\limits_{{k = 1}}^{j} {\boldsymbol{v}^{{(k)}} } \left[ {\left( {\boldsymbol{v}^{{(k)}} } \right)^{T} \boldsymbol{v}} \right]$. By definition of the directions ***v***^(*j*)^, we have *F*_*v*_(***L***^(*i*)^(***x***^(*i*)^ + ***v***^(1)^),***R***(***x***^(*i*)^)) > *F*_*v*_(***L***^(*i*)^(***x***^(*i*)^ + ***v***^(2)^),***R***(***x***^(*i*)^)) > … > *F*_*v*_(***L***^(*i*)^(***x***^(*i*)^ + ***v***^(*n*)^),***R***(***x***^(*i*)^)). The majority of antenna response variability is associated with the first few directions. For the sake of quantification, we consider the coefficients *C*_*j*_

20 $$C_{j} = \frac{{\sqrt {\sum\nolimits_{{k = 1}}^{j} {\left[ {F_{v} (\boldsymbol{L}^{{(i)}} (\boldsymbol{x}^{{(i)}} + \boldsymbol{v}^{{(k)}} ),\boldsymbol{R}(\boldsymbol{x}^{{(i)}} ))} \right]} ^{2} } }}{{\sqrt {\sum\nolimits_{{k = 1}}^{n} {\left[ {F_{v} (\boldsymbol{L}^{{(i)}} (\boldsymbol{x}^{{(i)}} + \boldsymbol{v}^{{(k)}} ),\boldsymbol{R}(\boldsymbol{x}^{{(i)}} ))} \right]} ^{2} } }}$$

The FD-based sensitivity update will be restricted to *N*_*update*_ directions computed as

21 $$N_{{update}} = \arg \mathop {\min }\limits_{{}} \left\{ {j \in \{ 1,2,...,n\} :C_{j} \ge C_{{th}} } \right\}$$

where *C*_*th*_ is the user-defined threshold, e.g., 0.90 (see^100^ for more extensive discussion). The details of Jacobian updating procedure using {***v***^(*j*)^}_*j* = 1, …, *Nupdate*_, can be found in Table. 2.

**Table 2:** Sensitivity updating procedure using principal directions.

Comment: The updating process cannot be based on rudimentary FD as the directions ***v***^(*j*)^ are generally not aligned with the coordinate system axes. Here, it is implemented using the rank-one Broyden formula^100^ , which allow for incorporating the antenna response data in arbitrary direction Sensitivity updating procedure: 1. Set *j* = 1; 2. Calculate a temporary point ***x***_*tmp*_ = ***x***^(*i*)^ + *h****v***^(*j*)^, where *h* > 0 is the step size; 3. Obtain EM-simulated antenna responses ***R***(***x***_*tmp*_); 4. Update the sensitivity matrix (here, ***h***^(*j*)^ = *h****v***^(*j*)^): $J_{R} (x^{{\left( i \right)}} )\;\; < = \;\;J_{R} (x^{{\left( i \right)}} ) + \frac{{\left( {\left[ {R(x_{{tmp}} ) - R(x^{{\left( i \right)}} )} \right] - J_{R} (x^{{\left( i \right)}} ) \cdot h^{{\left( j \right)}} } \right) \cdot h^{{\left( i \right)T}} }}{{h^{{\left( j \right)T}} h^{{\left( j \right)}} }}$; **5. If** *j* < *N*_*update*_ Go to 2; **else** END **end**
Remark: The step size h is set to a small fraction of mm (e.g., 0.02 to 0.1), which is a typical value for FD realized on EM-simulated antenna responses

### Complete optimization procedure

The complete optimization procedure is summarized here. The input to the algorithm includes the design space *X*, EM models (low-resolution ***R***_*c*_ and high-resolution ***R***_*f*_), target operating parameter vector ***f***_*t*_, operating performance vector definitions (***f*** and ***l***), and formulation of the objective function *U*_*L*_ (cf. (9)). Among these, ***f***_*t*_ and *U*_*L*_ depend on the design specifications, whereas the remaining parameters are antenna specific.

**Fig. 3:**
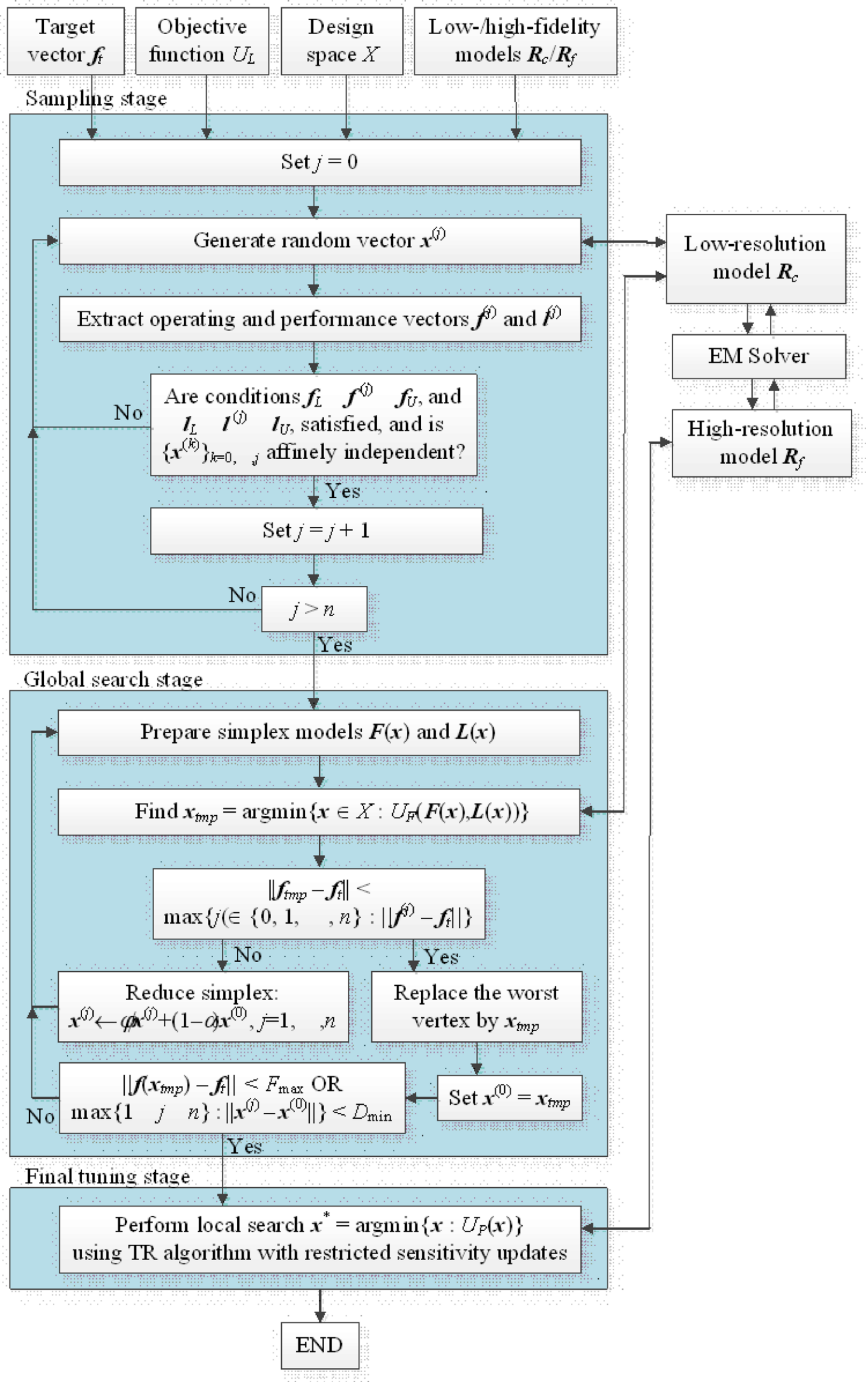
Flow diagram of the presented procedure for globalized antenna optimization using variable-fidelity EM models, simplex-driven regression models and rapid local tuning by means of principal directions.

The algorithm control parameters have been juxtaposed in Table 3. Still, the values of those parameters are not critical, as most of them mainly control the search process resolution. An exception is *F*_max_. As discussed earlier (Sect. 2.4.2), its value should be set up, specifically by accounting for the antenna operating bandwidths. The flow diagram of the presented framework is showcased in Fig. 3.

**Table 3:** Control parameters and their recommended values.

Parameter /default value	Explanation
*F*_max_	Termination threshold for the global search (specific to the considered problem, usually, one tenth to one fifth of GHz)
*α* = 0.2	Search region extension factor
*γ* = 0.5	Simplex reduction ratio
*D*_min_	Minimum simplex size, for which the global search stage is terminated (typically, 1% of the design space extent)
*ε* = 10^–3^	Termination condition for the local refinement
*C*_*th*_ = 0.9	Response variability threshold for gradient refinement via principal directions

It is important to emphasize conceptual differences between the proposed approach and the mainstream techniques reported in the literature. Leaving alone direct EM-driven global optimization, which is typically prohibitive in computational terms, global search is normally carried out using some sort of surrogate model. The existing approaches can be categorized into three main groups listed in Table 4: (i) surrogate-assisted methods where the metamodel is constructed upfront, then used for optimization purposes, (ii) machine learning methods where the surrogate is iteratively refined and acts as a fast predictor generating candidate solutions (infill points), and (iii) surrogate-aided bio-inspired algorithms. For comparison, Table 4 also provides the main conceptual points of the proposed technique. It can be observed that our approach is entirely different in terms of the methodology and the applied algorithmic tools. The practical advantages resulting from adopting these tools will be highlighted in Sect. 2.

**Table 4:** Available global search techniques and the proposed approach: methodological comparison.

Reference	Methodology		Comments
^60, 65-67, 69^	Surrogate model constructed as a separate stage, then applied to carry out the optimization process		Typical modeling methods: kriging, Gaussian process regression, neural networks
^70,72^	Surrogate model constructed within the machine learning framework (iteratively updated using accumulated EM simulation data) and used as a predictor		Typical modeling methods: Gaussian process regression, kriging, neural networks
^76,77,96^	Surrogate model constructed within nature-inspired optimization		Typical modeling methods: support vector regression, neural networks
This work	Main components: • Two separate search stages • Global search at the level of operating parameters (and using a low-fidelity EM model) • Simplex-based regressors leveraging weakly nonlinear dependence between operating parameters and design variables • Accelerated fine-tuning using principal directions and dual-resolution EM analysis		Modeling approach: simplex-based regressors

## Demonstration studies and benchmarking

The algorithm outlined in Sect. 2 is demonstrated here by optimizing four microstrip antenna structures, and it is compared to several procedures, including population-based metaheuristics, random-start gradient-based tuning, and two surrogate-assisted techniques. The obtained results confirm the following features of the search process: ability to allocate globally optimal design, exceptional design quality, and cost efficacy. The test cases and experimental setup are described in Sect. 3.1 and 3.2. Section 3.3 summarizes the performance of the presented optimization procedure.

### Verification antennas

**Fig. 4:**
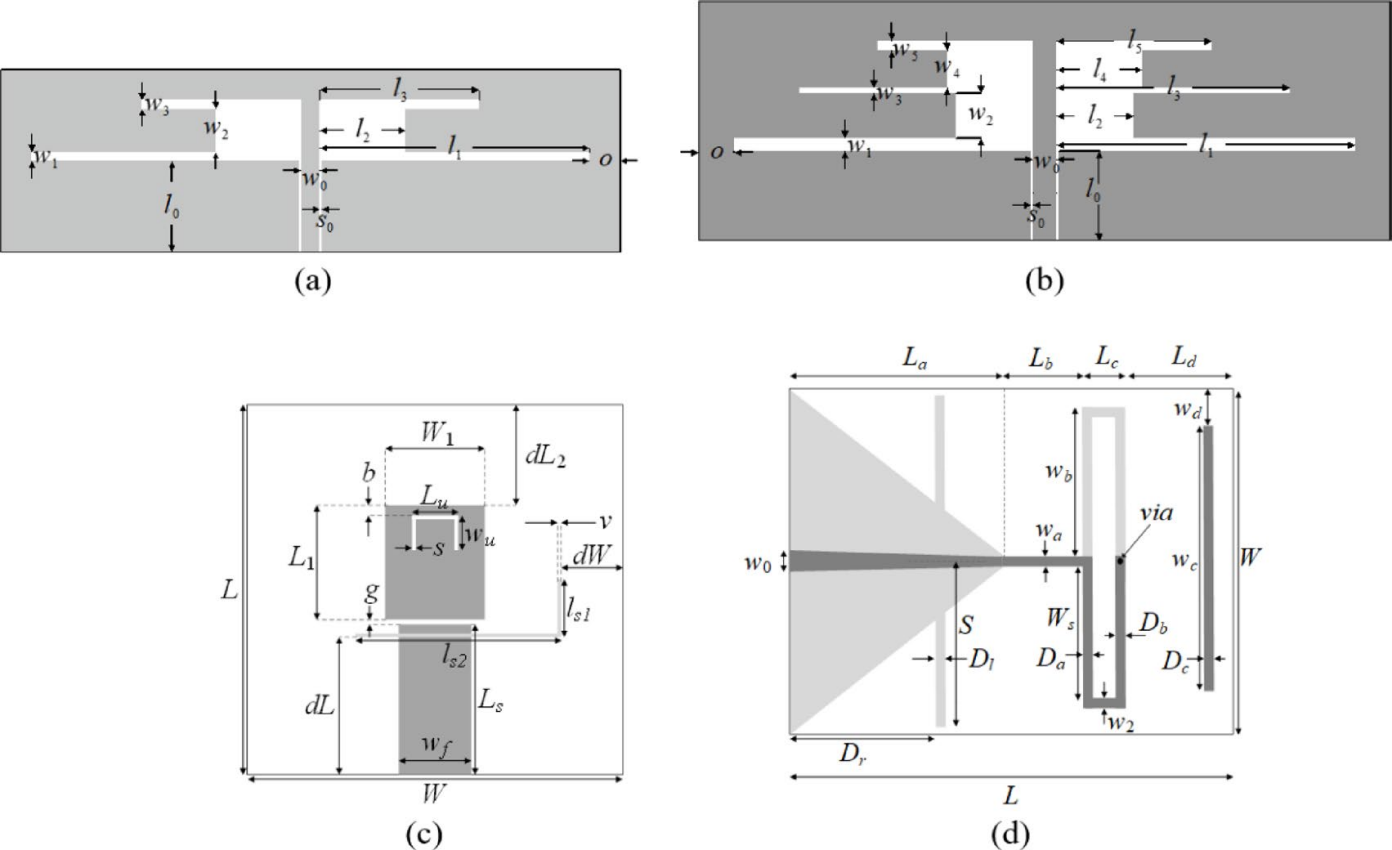
Verification test cases: (**a**) Antenna I^102^, (**b**) Antenna II^103^, (**c**) Antenna III^104^, the light-shade gray refers to a ground-plane slot, (**d**) Antenna IV^105^, the light-shade gray corresponds to ground plane.

**Fig. 5:**
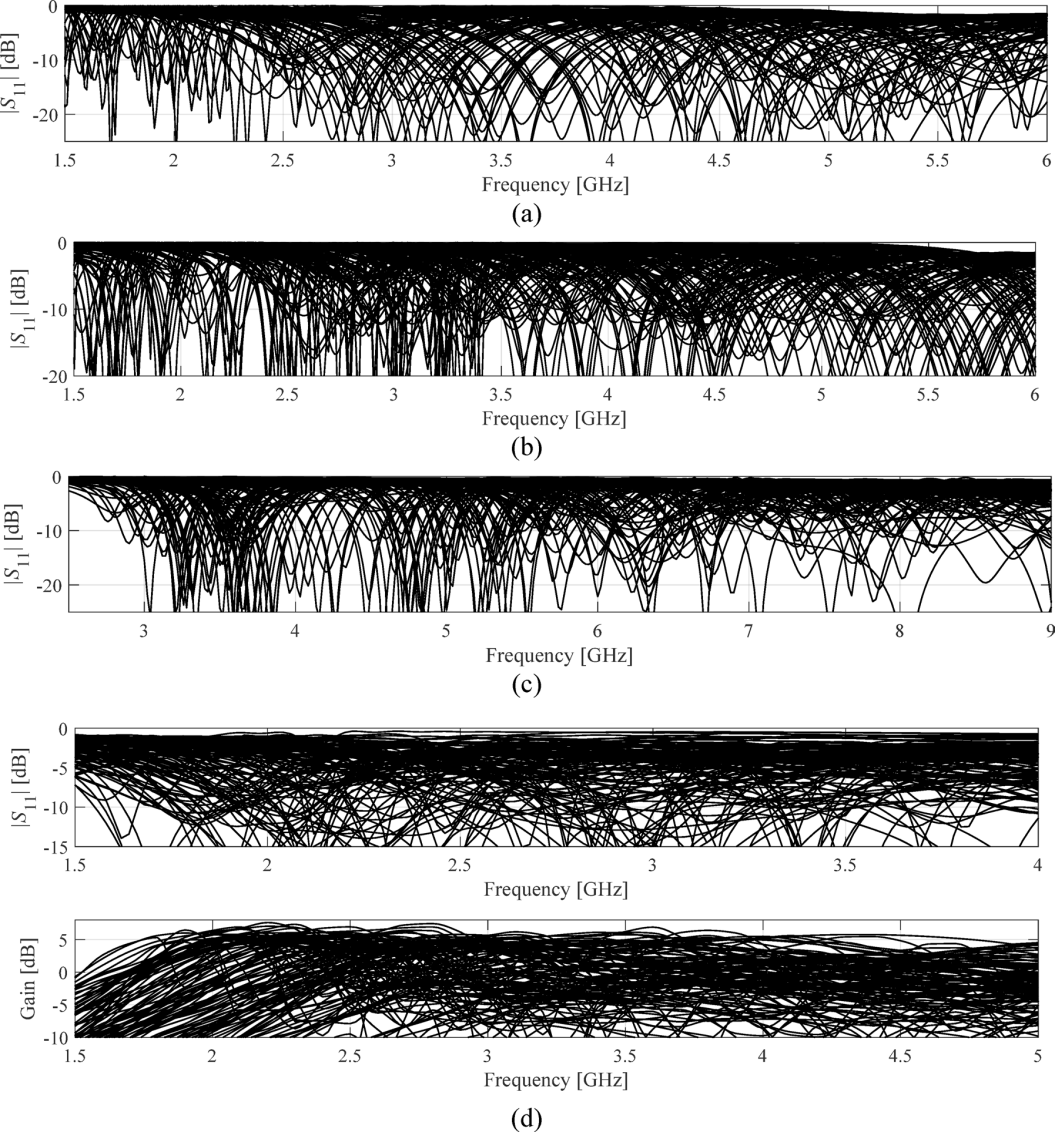
Randomly generated responses of the test antennas demonstrating significant variations of their responses within the assumed parameter spaces (100 designs generated for each structure): (**a**) Antenna I, (**b**) Antenna II, (**c**) Antenna III, (**d**) Antenna IV. The pictures corroborate a challenging nature of the considered verification problems.

The verification experiments are performed using four planar antennas illustrated in Fig. 4. The test set includes dual- and triple-band uniplanar dipole antennas (Antenna I^102^, Antenna II^103^, a triple-band U-slotted patch featuring a defected ground structure (Antenna III^104^, and a quasi-Yagi antenna with an integrated balun (Antenna IV)^105^. The detailed data on Antennas I through IV can be found in Fig. 5. The first three structures are optimized for best matching at the target operating frequencies (vector ***f***_*t*_). Antenna IV is designed for maximum in-band gain with the constraint imposed on the reflection response (|*S*_11_| ≤ − 10 dB within the antenna operating band). Electromagnetic analysis is conducted in CST Microwave Studio. The models of lowest fidelity are set up to ensure a sufficient rendition of all important properties of the antenna outputs (in particular, the resonances). The high-fidelity models are obtained through the grid convergence study. The resolution level is adjusted using the lines-per-wavelength parameter of CST allowing to adjusting the antenna’s discretization level.

**Fig. 6:**
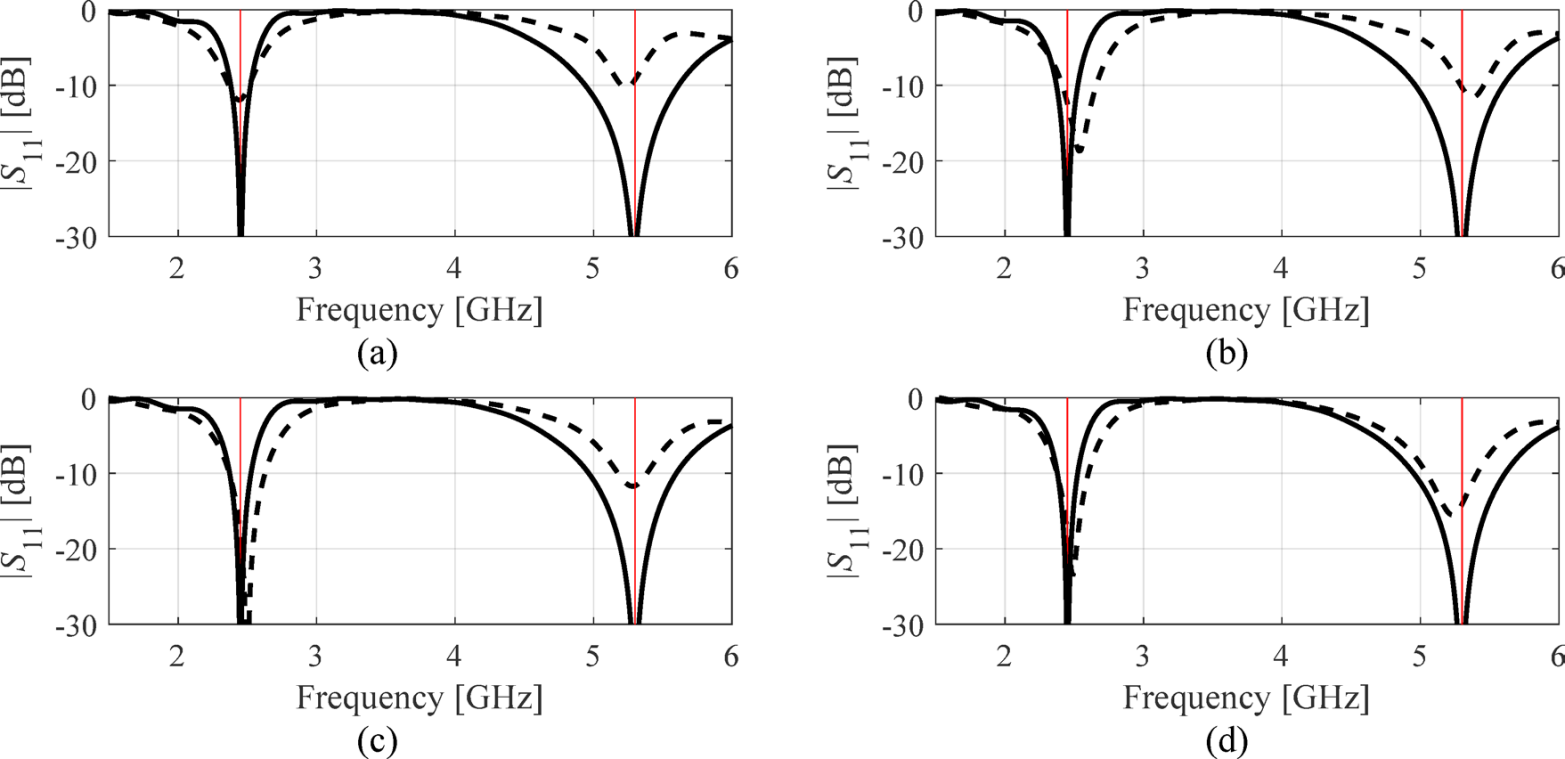
Antenna I: |*S*_11_| at the selected designs identified by the presented procedure. The panels (**a**) through (**d**) show algorithm runs 1 through 4. The global search solution ***x***^(0)^ is marked with dashed line, the final refined design is marked using solid line. The target operating frequencies (2.45 GHz and 5.3 GHz) are shown using vertical lines.

It should be noted that the dimensionality of the design spaces (from 6 to 14), broad ranges of parameters (the average ratio between upper and lower parameter bounds ranging from three to eight), and evaluation frequency (e.g., 0.5 GHz to 10 GHz for Antenna III) make the considered optimization problems challenging. Figure 6 shows randomly generated responses of Antennas I through IV (100 random designs for each case), demonstrating that the frequency characteristics vary significantly within the assumed parameter spaces.

### Experimental setup

The framework introduced in Sect. 2 has been used to optimize the test antennas of Fig. 4. The default control parameter setup has been used in all cases (*F*_max_ = 0.2 GHz, *α* = 0.2, *γ* = 0.5, *D*_min_ = 1, *ε* = 10^–3^, *C*_*th*_ = 0.9, cf. Table 5 to demonstrate no need for algorithm tuning to a particular problem at hand. Our methodology has been compared to the techniques listed in Table 6. The arrangement of the comparison pool allows us to verify the importance of each algorithmic mechanism embedded into our algorithm. Benchmarking against PSO (Algorithm I) and DE (Algorithm II) illustrates the computational speedup over population-based methods. It is noteworthy that although PSO and DE are run with a low budget of up to 1,000 objective function calls, they are still considerable on absolute terms (a few days of CPU time). A comparison with random-start gradient search (Algorithm III) demonstrates the multimodality of the considered verification case studies. Meanwhile, including Algorithms IV and V enables verification of the computational benefits associated with employing variable-resolution EM simulations and restricted sensitivity updates using principal directions. At the same time, it allows us to investigate whether these means degrade the quality of the antenna designs rendered in the optimization process.

**Table 5:** Technical data on considered antennas.

Parameter	Antenna structure			
	Antenna I^101^	Antenna II^102^	Antenna III^103^	Antenna IV^104^
Substrate	RO4350 (*ε*_*r*_ = 3.5, *h* = 0.76 mm)	RO4350 (*ε*_*r*_ = 3.5, *h* = 0.76 mm)	*ε*_*r*_ = 3.2 *h* = 3.0 mm	RO4003 (*ε*_*r*_ = 3.38, *h* = 1.5 mm)
Design parameters^$^	***x*** = [*l*_1_ *l*_2_ *l*_3_ *w*_1_ *w*_2_ *w*_3_]^*T*^	***x*** = [*l*_1_ *l*_2_ *l*_3*r*_* l*_4_ *l*_5*r*_* w*_1_ *w*_2_ *w*_3_ *w*_4_ *w*_5_]^*T*^	***x*** = [*L*_1_ *L*_*s*_* L*_*ur*_* W W*_1_ *dL*_*r*_* dW*_*r*_* g l*_*s*1*r*_* l*_*s*2*r*_ *w*_*ur*_]^*T*^	***x*** = [*L*_*a*_* L*_*b*_* L*_*c*_* L*_*d*_* W w*_*a*_* D*_*a*_* D*_*b*_* D*_*c*_* D*_*lr*_* D*_*rr*_* S*_*r*_* w*_*br*_* w*_*cr*_]^*T*^
Other parameters^$^	*l*_0_ = 30, *w*_0_ = 3, *s*_0_ = 0.15, *o* = 5	*l*_3_ = *l*_3*r*_*l*_1_ and *l*_5_ = *l*_5*r*_*l*_3_; *l*_0_ = 30, *w*_0_ = 3, *s*_0_ = 0.15, *o* = 5	*L* = *L*_*s*_ + *g* + *L*_1_ + *dL*_2_, *L*_*u*_ = *L*_*ur*_*W*_1_, *dL* = *dL*_*r*_*L*, *dW* = *dW*_*r*_*W*, *l*_*s*1_ = *l*_*s*1*r*_(*L* – *dL*), *l*_*s*2_ = *l*_*s*2*r*_(*W* – *dW*), *w*_*u*_ = *w*_*ur*_(*L*_1_ – *b* – *s*)	*D*_*l*_ = *D*_*lr*_*L*_*a*_, *D*_*r*_ = *D*_*rr*_*L*_*a*_, *S* = *S*_*r*_*W*, *w*_*b*_ = *w*_*br*_*W*/2, *w*_*c*_ = *w*_*cr*_*W*, *w*_0_ = 3.4
EM model	CST Microwave Studio	CST Microwave Studio	CST Microwave Studio	CST Microwave Studio
Low-resolution model ***R***_*c*_	~ 60,000 mesh cells Simulation time 25 s	~ 71,000 mesh cells Simulation time 35 s	~ 120,000 mesh cells Simulation time 35 s	~ 81,000 mesh cells Simulation time 39 s
High-resolution model ***R***_*f*_	~ 410,000 mesh cells Simulation time 92 s	~ 270,000 mesh cells Simulation time 80 s	~ 750,000 mesh cells Simulation time 182 s	~ 550,000 mesh cells Simulation time 150 s
Target operating frequencies [GHz]	***f***_*t*_ = [2.45 5.3]^*T*^	***f***_*t*_ = [2.45 3.6 5.3]^*T*^	***f***_*t*_ = [3.5 5.8 7.5]^*T*^	***f***_*t*_ = 2.5
Design goals	Minimize reflection at all operating frequencies	Minimize reflection at all operating frequencies	Minimize reflection at all operating frequencies	Maximize realized gain in ± 50 MHz bandwidth *B* centred at ***f***_*t*_; Constraint: |*S*_11_|≤ –10 dB at the same bandwidth
Primary objective function *U*(x)	*U*(***x***) = max{***x*** : max{|*S*_11_(***x***,*f*_*t.*1_)|,…, |*S*_11_(***x***,*f*_*t.K*_)|}}			*U*(***x***) = –*F*^–1^_*F*_*G*(***x***,*f*)*df*, *F* = [*f*_*t*_ – *B*/2, *f*_*t*_ + *B*/2]
Parameter space *X*	***l*** = [15 3 0.35 0.2 1.8 0.5]^*T*^ ***u*** = [50 12 0.85 1.5 4.3 2.7]^*T*^	***l*** = [20 3 0.6 3 0.6 0.2 0.2 0.2 0.2 0.2]^*T*^ ***u*** = [50 5 0.85 5 0.85 2.2 4.2 2.2 4.2 2.2]^*T*^	***l*** = [10 17 0.2 45 5 0.4 0.15 0.2 0.1 0.5 0.1]^*T*^ ***u ***= [16 25 0.6 55 15 0.5 0.3 0.8 0.4 0.65 0.5]^*T*^	***l*** = [15 5 1 15 25 0.5 1 1.5 1.5 0.05 0.4 0.5 0.5 0.5]^*T*^ ***u*** = [35 25 8 40 60 2.5 3.0 4.5 4.5 0.25 0.9 1.0 1.0 1.0]^*T*^

^$^ Dimensions in mm, except relative one (with subscript *r*), which are unitless.

**Table 6:** Setup of the proposed and benchmark procedures.

Algorithm	Algorithm type	Setup
This work	Variable-resolution simplex-based global search with gradient-based fine-tuning and sparse sensitivity updates (Section "Globalized Search by Regression Predictors, Variable-Resolution Models, and Restricted Sensitivity Updating")	Control parameters: *F*_max_ = 0.2 GHz, *α* = 0.2, *γ* = 0.5, *D*_min_ = 1, *ε* = 10^–3^ and *C*_*th*_ = 0.9 (see Table 3 for explanation of terms)
I	Particle swarm optimizer (PSO)	Swarm size *N* = 10, standard control parameters (*χ* = 0.73, *c*_1_ = *c*_2_ = 2.05); nthe umber of iterations set to 50 (Version I) and 100 (Version II)
II	Differential evolution (DE)	Population size *N* = 10, number of iterations set to 50 (Version I) and 100 (Version II); conventional parameter setup (*CR* = 0.5, *F* = 1) ^75^;
III	Trust-region gradient-based optimizer^97^	Random initial design, response gradients estimated using finite differentiation, termination criteria based on convergence in argument, and reduction of the trust region size^97^
IV	Simplex-based global search with gradient-based fine-tuning: single-resolution, no local tuning acceleration	Algorithm setup: similar to that of the proposed approach, except that the entire search process is conducted at the level of a high-resolution EM model, whereas local tuning employs a basic version of the TR algorithm (no acceleration)
V	Simplex-based global search with gradient-based fine-tuning: variable-resolution, no local tuning acceleration	Algorithm setup: the same as for the proposed approach, except that local tuning employs a basic version of the TR algorithm (no acceleration)

### Results

**Fig. 7:**
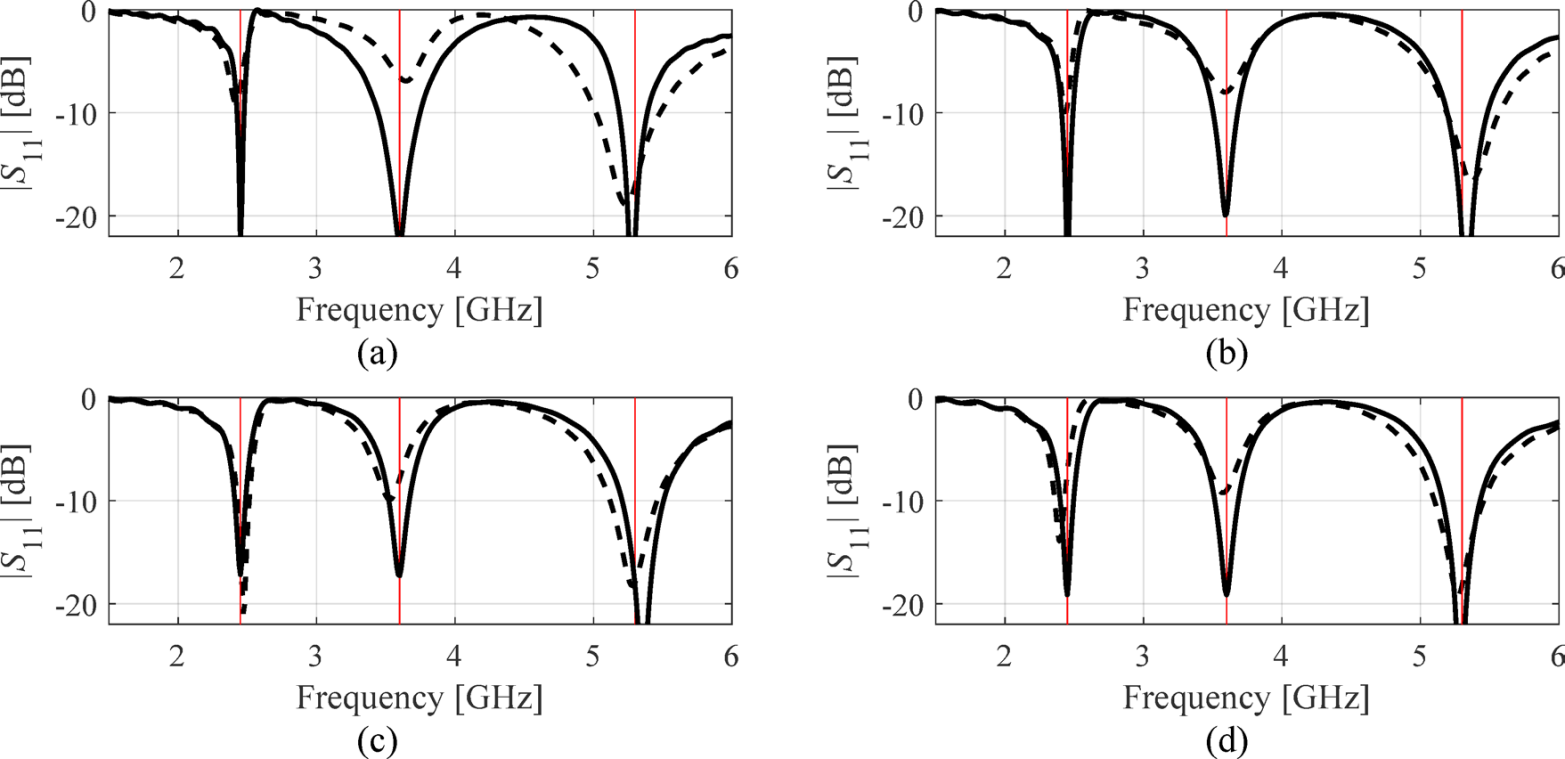
Antenna II: |*S*_11_| at the selected designs identified by the presented procedure. The panels (**a**) through (**d**) show algorithm runs 1 through 4. The global search solution ***x***^(0)^ is marked with dashed line, the final refined design is marked using solid line. The target operating frequencies (2.45 GHz, 3.6 GHz, and 5.3 GHz) are shown using vertical lines.

**Fig. 8:**
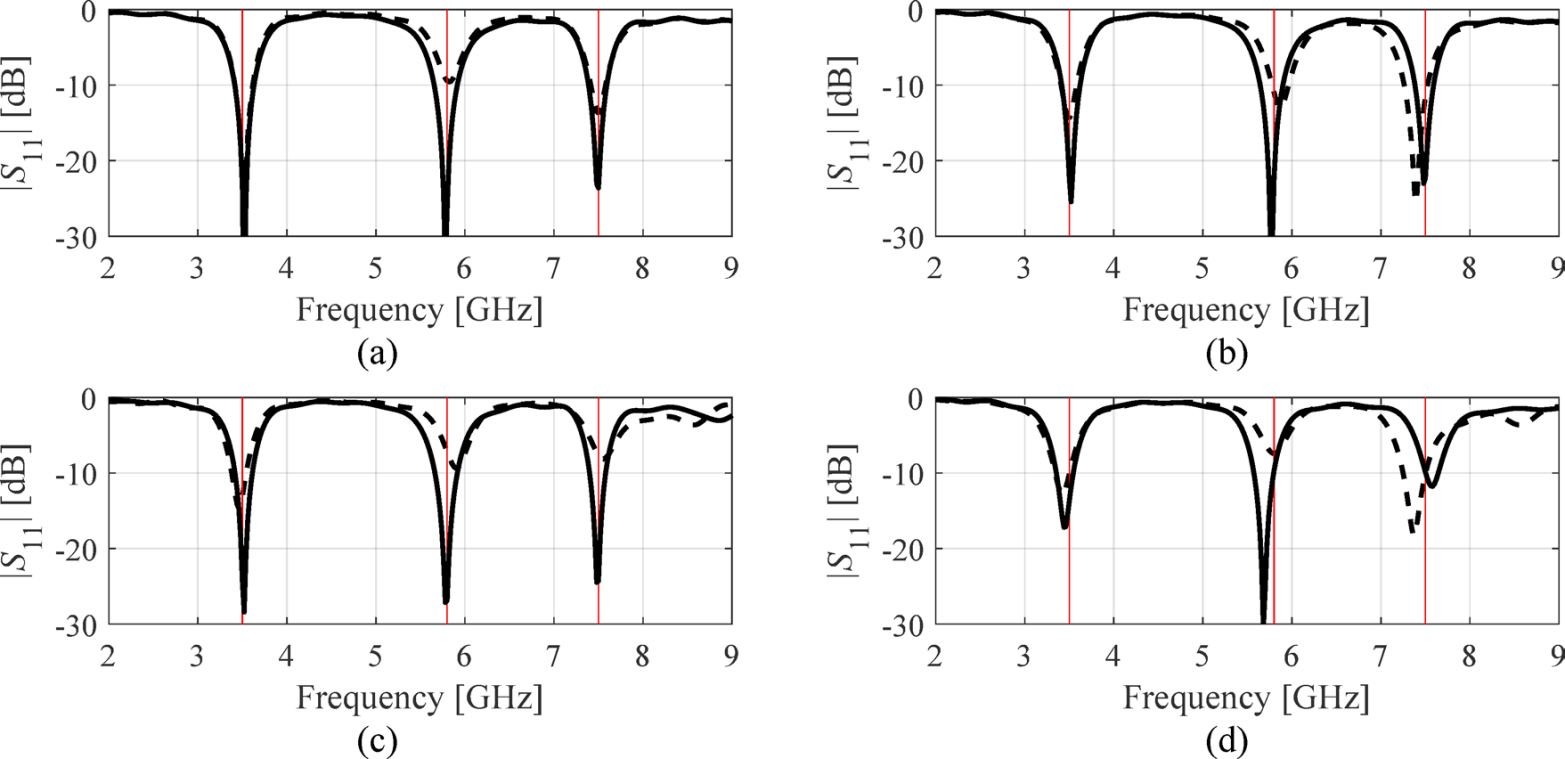
Antenna III: |*S*_11_| at the selected designs identified by the presented procedure. The panels (**a**) through (**d**) show algorithm runs 1 through 4. The global search solution ***x***^(0)^ is marked with dashed line, the final refined design is marked using solid line. The target operating frequencies (3.5 GHz, 5.8 GHz, and 7.5 GHz) are shown using vertical lines.

**Fig. 9:**
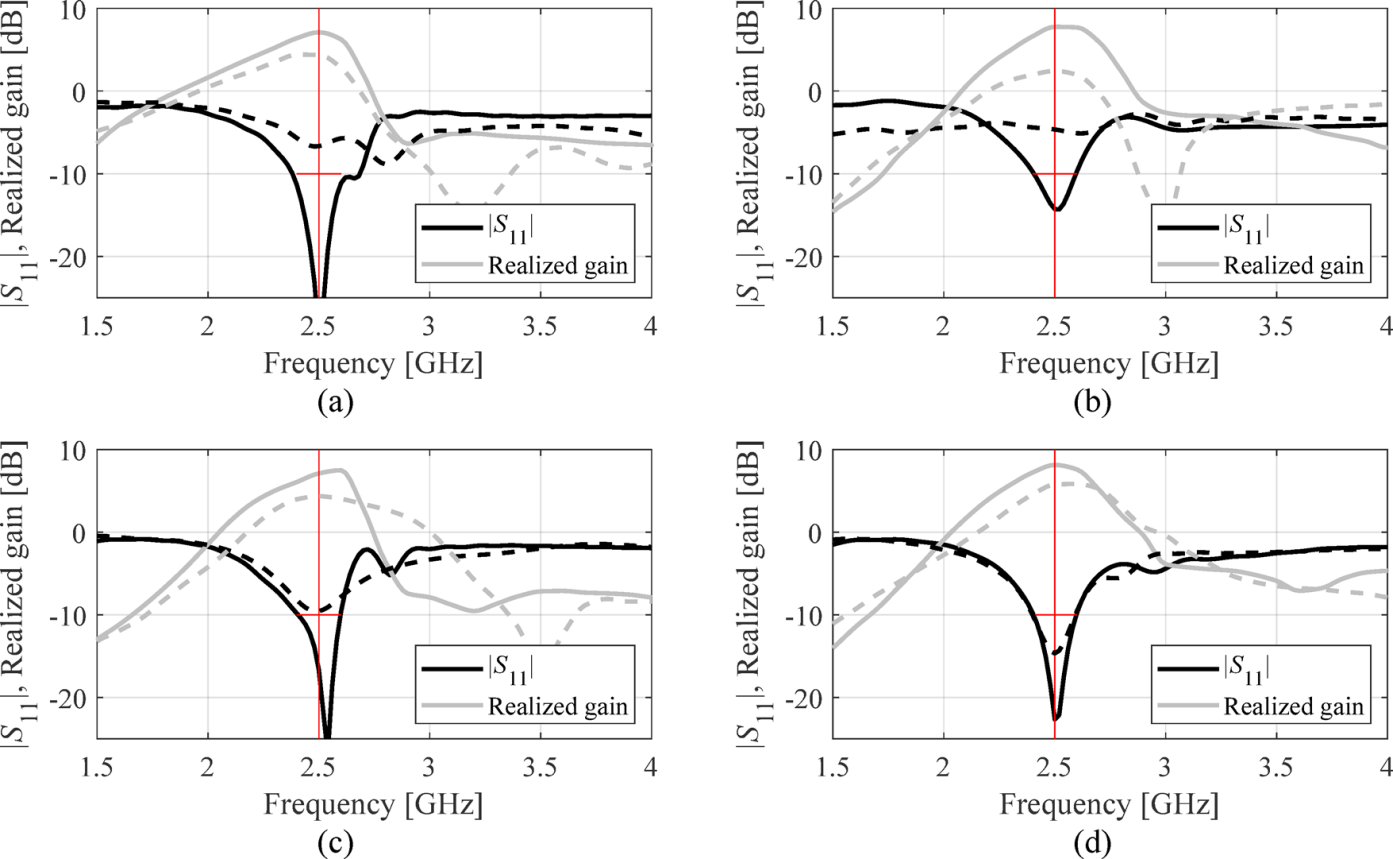
Antenna IV: |*S*_11_| and realized gain at the selected designs identified by the presented procedure. The panels (**a**) through (**d**) show algorithm runs 1 through 4. The global search solution ***x***^(0)^ is marked with dashed line, the final refined design is marked using solid line. The target operating frequency (2.5 GHz) is shown using vertical line. The horizontal line marks the intended impedance matching bandwidth (at the level of − 10 dB).

 Table 7, Table 8, Tables 9, and Table 10 put together the results. Antenna reflection at the solutions found in selected executions of the presented framework have been included in Figs. 6, 7 and 8, and Fig. 9 for antennas I, II, III, and IV. Ten independent optimization runs have been executed for each antenna structure using the proposed algorithm and the benchmark. The reported data represents the average values of the objective function along with the computational expenses. The latter is calculated based on the number of EM analyses evaluated using the highest resolution.

A critical performance factor is the success rate, which quantifies the number of optimization instances where the operating parameters have been relocated into the vicinity of the target. In the following, we analyze the operation of the introduced approach concerning the cost efficiency and the optimization process dependability, and how it compares to the benchmark methods.

**Table 7:** Antenna I: optimization results.

Optimization method		Average objective function value [dB]	Computational cost^$^	Success rate^#^
Variable-resolution simplex-based algorithm with restricted sensitivity updates (this work)		–31.0	56.0	10/10
Algorithm I (PSO)	Version I (50 iterations)	–18.2	500	9/10
	Version II (100 Iterations)	–19.3	1,000	10/10
Algorithm II (DE)	Version I (50 iterations)	–21.5	500	9/10
	Version II (100 Iterations)	–22.8	1,000	9/10
Algorithm III (Trust-region gradient-based algorithm)		–13.5	84.2	6/10
Algorithm IV (Simplex-based algorithm with high-fidelity models; no local tuning acceleration)		–25.3	82.9	10/10
Algorithm V (Simplex-based algorithm with multi-fidelity models, no local tuning acceleration)		–30.9	65.3	10/10

^$^ The cost is assessed w.r.t. the number of equivalent high-resolution EM analyses of the considered antenna structure. It is computed by dividing the total EM simulation time (all EM analyses, including the low- and high-fidelity models, executed during the optimization process) by the simulation time of the high-fidelity model.

^#^ Number of algorithms executions ensuring ||***f***(x^*^) – ***f***_*t*_|| < *F*_max_ (i.e., the center frequencies are adequately aligned with the targets).

**Table 8:** Antenna II: optimization results.

Optimization method		Average objective function value [dB]	Computational cost^$^	Success rate^#^
Variable-resolution simplex-based algorithm with restricted sensitivity updates (this work)		–19.8	111.5	10/10
Algorithm I (PSO)	Version I (50 iterations)	–10.8	500	5/10
	Version II (100 Iterations)	–13.8	1,000	8/10
Algorithm II (DE)	Version I (50 iterations)	–12.1	500	6/10
	Version II (100 Iterations)	–13.6	1,000	8/10
Algorithm III (Trust-region gradient-based algorithm)		–7.8	105.8	4/10
Algorithm IV (Simplex-based algorithm with high-fidelity models; no local tuning acceleration)		–17.5	154.0	10/10
Algorithm V (Simplex-based algorithm with multi-fidelity models, no local tuning acceleration)		–20.1	122.0	10/10

^$^ The cost is assessed w.r.t. the number of equivalent high-resolution EM analyses of the considered antenna structure. It is computed by dividing the total EM simulation time (all EM analyses, including the low- and high-fidelity models, executed during the optimization process) by the simulation time of the high-fidelity model.

^#^ Number of algorithms executions ensuring ||***f***(x^*^) – ***f***_*t*_|| < *F*_max_ (i.e., the center frequencies are adequately aligned with the targets).

**Table 9:** Antenna III: optimization results.

Optimization method		Average objective function value [dB]	Computational cost^$^	Success rate^#^
Variable-resolution simplex-based algorithm with restricted sensitivity updates (this work)		–20.3	55.5	10/10
Algorithm I (PSO)	Version I (50 iterations)	–12.3	500	6/10
	Version II (100 Iterations)	–14.2	1,000	8/10
Algorithm II (DE)	Version I (50 iterations)	–13.5	500	7/10
	Version II (100 Iterations)	–15.1	1,000	9/10
Algorithm III (Trust-region gradient-based algorithm)		–12.1	125.4	4/10
Algorithm IV (Simplex-based algorithm with high-fidelity models; no local tuning acceleration)		–17.5	110.7	10/10
Algorithm V (Simplex-based algorithm with multi-fidelity models, no local tuning acceleration)		–20.7	81.1	10/10

^$^ The cost is assessed w.r.t. the number of equivalent high-resolution EM analyses of the considered antenna structure. It is computed by dividing the total EM simulation time (all EM analyses, including the low- and high-fidelity models, executed during the optimization process) by the simulation time of the high-fidelity model.

^#^ Number of algorithms executions ensuring ||***f***(x^*^) – ***f***_*t*_|| < *F*_max_ (i.e., the center frequencies are adequately aligned with the targets).

**Table 10:** Antenna IV: optimization results.

Optimization method		Average objective function value [dB]^&^	Computational cost^$^	Success rate^#^
Variable-resolution simplex-based algorithm with restricted sensitivity updates (this work)		7.6	89.4	10/10
Algorithm I (PSO)	Version I (50 iterations)	6.1	500	9/10
	Version II (100 Iterations)	6.8	1,000	10/10
Algorithm II (DE)	Version I (50 iterations)	6.0	500	8/10
	Version II (100 Iterations)	6.9	1,000	10/10
Algorithm III (Trust-region gradient-based algorithm)		–1.1	138.4	1/10
Algorithm IV (Simplex-based algorithm with high-fidelity models; no local tuning acceleration)		7.4	144.3	10/10
Algorithm V (Simplex-based algorithm with multi-fidelity models, no local tuning acceleration)		7.6	115.1	10/10

^&^The reported values correspond to the realized gain at 2.5 GHz (target operating frequency).

^$^ The cost is assessed w.r.t. the number of equivalent high-resolution EM analyses of the considered antenna structure. It is computed by dividing the total EM simulation time (all EM analyses, including the low- and high-fidelity models, executed during the optimization process) by the simulation time of the high-fidelity model.

^#^ Number of algorithms executions ensuring ||***f***(x^*^) – ***f***_*t*_|| < *F*_max_ (i.e., the center frequencies are adequately aligned with the targets).

We start by investigating the dependability of the search process regarding the algorithm success rate. On the one hand, all presented problems are multimodal: the average success factor of random-start gradient optimization is as low as 4/10. On the other hand, the performance of nature-inspired optimization is mediocre (success rate of 7/10 for Version I, and 9/10 for Version II of both PSO and DE), which indicates insufficient computational budget. As mentioned earlier, the latter was set up to avoid excessive running times, which are still up to three days per run for the budget of one thousand function evaluations. The reliability of the proposed approach and Algorithms IV and V is much better, with a success rate of 10/10 for all four antenna structures. This is expected as these three methods share underlying global search mechanisms. However, the presented algorithm is cost efficient, which will be analysed in-depth later.

The average cost function value at antenna designs produced by our algorithm is comparable to those obtained using the simplex-based benchmark procedures (Algorithms IV and V), which demonstrate that the acceleration mechanisms incorporated in our technique does not affect negatively the quality of the optimization process. The differences, if any, are minor. At the same time, the quality considerably surpasses that of the remaining benchmark methods (Algorithms I, II, and III), which is partially related to the inferior success rate of these procedures.

Without a doubt, computational efficiency is the most significant advantage of the presented approach. The average optimization cost equals only 78 EM antenna analyses at the high resolution, which is as much as 30% less than the cost of a straightforward gradient-based search. This result is not surprising when one considers that our technique incorporates various acceleration mechanisms, and its local tuning starts from an already good design rendered at the global search stage. The average savings over Algorithm IV reach 37% and 19% over Algorithm V. This indicates that the employment of dual-resolution EM analysis and restricted gradient updates (at the local tuning step) has similar effects on the speedup. The most dramatic differences are in comparison with PSO and DE. Here, the savings (w.r.t. Version II, budget of 1,000 function calls) are as high as over 92%, corresponding to the multiplicative acceleration factor of thirteen. Note that this speedup would be even greater should the nature-inspired routines were executed with more realistic budgets.

The performance indicators discussed above make the proposed approach attractive for globalized antenna optimization compared to more conventional methodologies, especially population-based metaheuristic algorithms. Since the search process is not conducted by processing the complete antenna responses but at the level of appropriately estimated operating parameters, the developed procedure is especially suitable for multi-band antennas, high-gain structures, etc., where identifying the operating figures is straightforward. A significant practical advantage of the algorithm is that it low number of control parameters and essentially no need to tune them to a particular problem, as demonstrated by testing all four antenna structures under the same setup.

It should be emphasized that the test cases considered in this section were selected to represent typical design problems, which are often related to impedance matching (appropriate allocation of antenna resonances and ensuring their sufficient depth is often the most essential optimization task before handling other types of responses), and to be sufficiently challenging (e.g., multi-band antennas are more difficult to handle than single-band or broadband antennas). It should be noted that the proposed method is suitable for handling any type of response because weakly nonlinear dependence between operating parameters and design variables carries over from reflection to other characteristics (e.g., gain, efficiency). In fact, treating, e.g., gain, is easier as it is an integral parameter, and it exhibits smaller variations (as a function of design variables).

A possible limitation of the presented technique is that the sampling process to identify the initial simplex vertices (cf. Section 2.3) may become computationally inefficient in the case of vast parameter spaces. More specifically, most random samples may be rejected due to poor-quality or distorted responses, making extracting the antenna operating parameter infeasible. The risk of this situation can be significantly minimized by appropriately selecting the lower and upper bounds for parameters (e.g., based on engineering experience). On the other hand, very large spaces would be just as troublesome for any global optimization method as well.

To evaluate the effects of control parameters, in particular, *F*_max_, *α*, *γ*, and *D*_min_, additional experiments were conducted for the first test case (Antenna I). Table 11 puts together the results. As mentioned earlier, as most parameters control the resolution of the search process, their effect on the algorithm’s performance is rather minor. It is observed that the perfect success rate is maintained for all parameter variations. The variations in the objective function values are within the limit of ± 2dB, which is insignificant given the baseline (< − 30 dB). The computational cost varies within ± 5 high-fidelity EM analyses. Note that diminishing *F*_max_ and *D*_min_ is more demanding for the global search stage, which carries over to a slightly higher cost (the effect is flattened due to using dual-fidelity models). Meanwhile, the cost of fine-tuning is typically lower, so that the global cost is comparable. On the other hand, varying *α* has almost no effect as expected, whereas reducing *γ* generally leads to a faster convergence of the global search. This may jeopardize reliability and increase the cost of local tuning. In general, relaxing global search convergence criteria makes the local tuning step more demanding (and longer), but these two factors counterbalance each other so that the overall cost is comparable.

**Table 11:** Impact of control parameter setup (Antenna I).

Control parameter				Results		
*F*_max_	*α*	*γ*	*D*_min_	Objective function [dB]	Computational cost	Success rate
0.2	0.2	0.5	1	–31.0	56.0	10/10
0.2	0.2	0.33	2	–31.3	61.3	10/10
0.2	0.1	0.5	1	–29.5	55.1	10/10
0.2	0.1	0.33	2	–28.3	62.4	10/10
0.1	0.2	0.5	1	–31.5	64.2	10/10
0.1	0.2	0.33	2	–33.1	53.8	10/10
0.1	0.1	0.5	1	–27.4	63.2	10/10
0.1	0.1	0.33	2	–32.0	66.4	10/10

## Conclusion

This work aimed to introduce a low-cost and reliable technique for globalized parameter tuning of multi-band antennas. Our approach capitalizes on conducting the initial stages of the optimization process from the standpoint of the operating conditions of the considered antenna, using simplex-driven regression models. The latter has the effect of regularizing the merit function landscape. Thus, it facilitates the identification of the most encouraging parts of the design space. Further acceleration has been achieved by incorporating dual-resolution EM models and restricted gradient refinement at the local tuning stage. Comprehensive verification corroborates the technique’s efficacy with the optimization’s average computational cost corresponding to less than eighty EM antenna analyses. The aforementioned algorithmic tools equally contribute to the achieved speedup, which is close to 40% over the simplex-based search at a high-fidelity level and 20% over the variable-fidelity version thereof (not using sparse sensitivity updates). These savings are not detrimental to the reliability of the search process or the quality of the antenna designs rendered by the procedure. The multiplicative acceleration factor over the nature-inspired routines (here, PSO and DE) is as high as thirteen, even though these algorithms were run as a relatively thrifty budget of only 1,000 merit function calls. Another vital advantage of the discussed method is its simple setup and handling. The algorithm features few control parameters, and their specific values do not have to be tuned to the considered optimization task.

The underlying principle of the proposed method, i.e., carrying out the optimization at the level of the antenna operating parameters, dramatically contributes to its reliability and computational efficiency; however, it may also be a source of limitations. In the case of very large parameter spaces, the sampling process of Sect. 2.3, oriented towards the identification of the initial simplex vertices, may be impaired. Most random vectors would correspond to poor designs, for which the operating parameters could not be extracted. As a result, the CPU cost of the algorithm would increase. At the same time, in practice, the parameter spaces are set up using engineering insight and parametric studies, which typically prevent the aforementioned issues from emerging. The same problem would generally be detrimental to any global optimization technique. Having said that, the presented framework seems to be a suitable replacement for the modern global optimization techniques, especially in EM-driven design.

## Data Availability

The datasets generated during and/or analysed during the current study are available from the corresponding author on reasonable request. Contact person: anna.dabrowska@pg.edu.pl.
